# Spectral classification of short numerical exon and intron sequences

**DOI:** 10.1186/1471-2105-12-S11-A13

**Published:** 2011-11-21

**Authors:** Benjamin YM Kwan, Jennifer YY Kwan, Hon Keung Kwan

**Affiliations:** 1 Faculty of Medicine, University of Ottawa, Ottawa, Ontario K1H 8MS, Canada; 2 School of Medicine, Queen’s University, Kingston, Ontario K7L 3N6, Canada; 3 Department of Electrical & Computer Engineering, University of Windsor, Windsor, Ontario N9B 3P4, Canada

## Abstract

This research presents three new numerical representations for classifying short exon and intron sequences using discrete Fourier transform period-3 value. Based on the human genome, results indicate that the Complex Twin-Pair representation is attractive compared with other numerical representations and the approach has potential applications in genome annotation and read mapping.

## Background

Current methods for genome annotation focus on sequence similarity or motif matching to known genes and there is a need for a complementary or more effective approach. It is known that protein coding (exonic or C-G rich) regions exhibit a period-3 property which is less prominent in noncoding (intronic or A-T rich) regions. The boundary between these 2 regions becomes less apparent as sequence length becomes shorter. The period-3 property is likely due to the 3-base-length of codons. C-G rich content in coding regions is due to nonuniform codon usage. For spectral analysis of period-3 value, a nucleotide sequence has to be converted to a numerical sequence. The choice of numerical representation affects how well its biological properties can be preserved and reflected.

## Methodology

Based on exon and intron sequences downloaded from UCSC Genome Browser on Human (GRCh37/hg19) (http://genome.ucsc.edu/cgi-bin/hgText) using [1-3], the classification performance in precisions (%) were computed by applying the spectral analysis and thresholding of [4] to the following twelve numerical representation methods: 1. Integer Number; 2. Single Galois Indicator; 3. Paired Nucleotide Atomic Number; 4. Atomic Number; 5. Molecular Mass; 6. EIIP; 7. Paired Numeric; 8. Real Number; 9. Complex Number; 10. Complex Twin-Pair (C, G = -1; A, T = j); 11. Complex Bipolar-Pair Code I (C = -1; G = 1; A = j; T = -j); 12. Complex Bipolar-Pair Code II (C = -1; G = 1; A = -j; T = j). Methods 1-9 are specified in [4] and Methods 10-12 are new numerical representations. In simulations, two adjacent windows are overlapped by 3 bases.

## Results and conclusions

**Figure 1:**
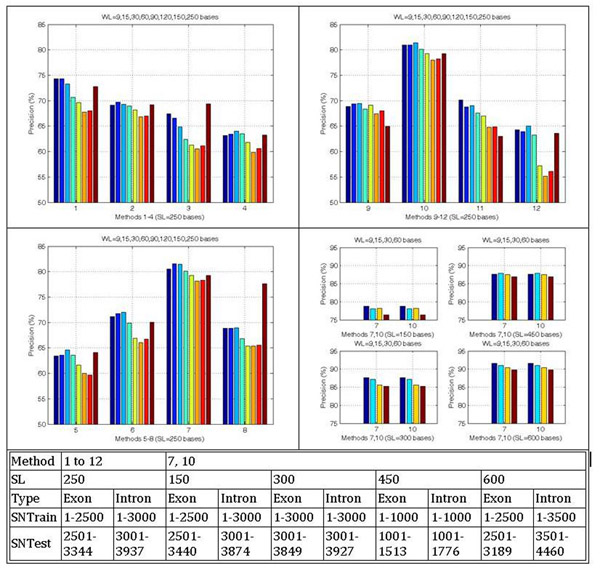
Precisions of methods obtained by different WL set and SL (SL: Sequence length in bases; WL: Window length in bases; SNTrain: Sequence numbers for training; SNTest: Sequence numbers for testing).

The results summarized in Figure 1 indicate that the approach is capable for effective classification of untrained short exon and intron sequences. Among the 3 new numerical representations, the Complex Twin-Pair (Method 10) achieves a precision of about 79% to 92% for a sequence length of 150 bases to 600 bases and a window length of 9 bases which is comparable with those of the Paired Numeric (Method 7).
